# New York Obstetrical Society

**Published:** 1885-03-20

**Authors:** 


### NEW YORK OBSTETRICAL SOCIETY.

### DYSTOCIA FROM AN ANOMALY OF THE FŒTAL SKULL.

Dr. Garrigues related a second case as follows:

The patient was an English lady, twenty-four years of age, whom he had seen in consultation. He found that she had been in labor for two days, but the os was only of the size of a silver dollar. He gave chloroform, introduced the hand into the vagina and dilated the os without difficulty. The patient was then allowed to rest a while to see if the pains would come on naturally. During the two preceding days there had been no true labor pains, although the patient had suffered all that time. Very little change taking place after dilatation, Dr. Garrigues put on the forceps, and, with the assistance of the family physician, labored for three hours in vain attempts to extract the head. The bones of the skull were so hard and the sutures so firm that they refused to yield in the least. The fontanelles were very small; the posterior one could be felt in the right occipito-posterior position. Although the woman's pelvis was well developed, it was perfectly evident that the child was too large and the bones of the head too greatly ossified to permit of delivery in the natural manner. The anterior fontanelle was then perforated, and the brain extracted without difficulty, but it was still impossible to make the slightest impression with the forceps. The cephalo-tribe was then applied, but it produced no cracking of the bones, and examination with the finger showed that the cranial bones had simply yielded like a plate of lead. The forceps was then re-applied, and, the cranioclast being used also, the head was finally extracted. Dr. Garrigues remarked that he had found this combination of the forceps and cranioclast useful also in another case. The head having started, he removed the forceps and finished delivery with Simpson's cranioclast alone. He had once attempted to use Braun's cranioclast, but was not favorably impressed with it. He had employed Simpson's twice, and had found it answer every desired purpose. Braun's was larger, and filled the entire vagina.

Some further difficulty was experienced in extracting the child's shoulders, but this was finally accomplished by means of the blunt hook. Dr. Garrigues' attendance on the woman had extended over twelve hours; the operation proper had lasted eight hours, during which time three ounces of chloroform and two pounds of ether were consumed. The placenta was removed by the introduction of two fingers into the womb and the use of deep pressure upon the abdomen. The uterus was washed out with a solution of bichloride of mercury, one part to two thousand, and his pad applied. The patient did well. On the second day the temperature rose to 102° F., and for a time there was a profuse purulent discharge from the wounds in the vagina, but there had been no further trouble.

The child's head and shoulders were very large. It weighed eleven pounds and a half, was twenty-two inches long, and the shoulder girth was seventeen inches. The cranial sutures were very narrow; the fontanelles exceedingly small. The bones were very thick and elastic, which accounted for the fact that the cephalotribe made no more impression upon them than if they had been composed of a thick plate of lead.

Dr. Henry Griswold (present by invitation) said he had seen a case two years ago similar to the first one related by Dr. Garrigues. The woman seemed to be perfectly well developed in every respect, but there was absence of the vagina, uterus and ovaries. There was a depression of the depth of a little over two inches where the vagina should have been. The woman had sexual desire, but did not menstruate. She had, however, the monthly molimen.

Dr. Hunter said that two cases parallel to that ralated by Dr. Garrigues had been under observation at the Woman's Hospital some years ago, in the service of Dr. Emmett. Both women were married, and the husband of each of them desired that an operation should be performed. Dr. Emmett made an artificial vagina about three inches in length, put in glass plugs, and kept the patients under observation a number of weeks. The tendency to reconstruction was very great, and the first patient returned after a year in about her original condition. The other patient returned with firm contraction of the canal at the end of about six months. Dr. Hunter was of the impression that the formation of an artificial vagina in cases in which there was no uterus would not prove of much benefit. It was exceedingly difficult to keep it open.

Dr. John Scott, of San Francisco (present by invitation), referred to the case of a well-developed woman in whom an artificial vagina had been made, and who was then living happily with her husband. No trace of a uterus or ovaries could be found. In another case the patient was willing to pay any sum of money if he could assure her that the uterus was present, but, on learning that the organ was absent, she committed suicide.

Dr. Van Ness (present by invitation) said a girl, eighteen years of age, had applied for admission to the Woman's Hospital, having suffered greatly from her first menstruation, which had recently taken place. Thus far, physical examination had revealed only a short cul-de-sac, without any signs of a uterus or ovaries. The fact that the patient had menstruated, however, would point to some trace of a uterus. There was now in the hospital a woman forty-five years of age, married twenty years, who had never had connection. Only a small cul-de-sac could be discovered at the situation of the vagina. There was now a growth of some sort in the pelvic cavity as large as a child's head at term, presumably a fibroid.

Dr. Colin Mackenzie said a young lady had been brought to him within the past six months, having never menstruated, and, on examination, no uterus or ovaries were to be found. Dr. Emmett, who saw the patient, said no operation was justifiable.

Dr. J. W. S. Gouley (present by invitation) said he had reported a case in the New York Medical Journal, in 1866 or 1867, in which there was absence of the uterus and ovaries. The patient suffered a great deal from headache.

The Chairman inquired whether the pelvis had been measured in Dr. Garrigues' case.

Dr. Garrigues replied that he had not measured the pelvis, but he supposed, from the examination and from the general appearance of the woman, that it was perfectly normal in size.

The Chairman said that the inference certainly would be that the pelvis was smaller than normal, for where there was absence of any of the pelvic contents there should be a lack of development of the pelvis itself. This was so commonly the rule that, when he found a congenital defect of the rectum with a relatively small pelvis, he inferred that there was a large piece of intestine wanting. He had observed this fact in several cases. If the pelvis was small in cases of congenital absence of the intestine, he thought it would also be found to be small in cases of absence of the sexual organs. It was true, however, that in the newly born the colon was much larger comparatively than at a more advanced age. Judging by analogy, he would not suppose that an artificial vagina would remain open without a transplantation of skin.

Dr. Garrigues remarked that the late Dr. Blake, of this society, thought a child with premature ossification of the bones of the cranium had a tendency to become idiotic, and that this fact should be taken into consideration when we were called upon to decide the question of sacrificing the child to accomplish delivery.

The Chairman was of the impression that in the case narrated by Dr. Garrigues there was not that degree of ossification which would prevent brain development. A great deal, however, would have to be left to the judgment of the physician in determining what procedure to adopt in such cases.

### CÆSAREAN SECTION.

Dr. Murray (present by invitation) reported a case which he had seen in consultation. The woman, aged thirty-two, had been in labor for thirty hours, attended by a midwife, before her physician was called. He found, on his arrival, that there was some deformity of the pelvis, but was unable to measure the superior strait on account of a large caput succedaneum. Simpson's forceps was applied to the head, but without success. When Dr. Murray was called the patient was almost moribund, the child's head was fixed, and the position was such that the diameters of the pelvis could not be taken. Perforation was performed, but it was still impossible to extract the child, and it was found that the conjugate would admit only two fingers, while the breadth was more than that of the hand. After failing with Braun's cephalotribe, Dr. Murray said nothing more could be done than to perform the Cæsarean section, but this was not consented to until later. Turning was performed, but it was then impossible to engage the child in the superior strait. Dr. Gillette having been added to the consultation, the Cæsarean section was performed. Not sufficient hæmorrhage took place from the uterine wound to wet a single sponge. The woman lived for two days and a half, most of the time in a state of collapse, and died. The point to which Dr. Murray wished to call attention was the difficulty, under the circumstances, of making out the diameters of the superior strait. He also remarked that one of the gentlemen present had suggested laparo-elytrotomy, and this, probably, would have been the proper operation to perform had consent been given sufficiently early. Dr. Murray said that he had seen a similar case, the deformity, however, not being so marked, in which he was enabled to extract the child without difficulty after perforation.

### TAPPING THE PERITONÆUM FOR TYMPANITES.

Dr. Hunter related a case in which a patient was attacked with peritonitis three days after laparotomy, the temperature being controlled by the abdominal coil. There had been very little hæmorrhage during the operation, and no tube was left in the wound. The important feature in the case was the development of an excessive degree of tympanites, the distension being enormous. Long rectal tubes were passed by the anus, and much gas escaped, but still a great amount of distension remained and caused the patient much distress. By the twelfth day she was thought to be moribund. The pulse was over 170. Dr Hunter then punctured the abdomen in the median line with a large hypodermic needle, and immediately gas began to escape, and continued to escape for half an hour. The patient very soon recovered from her state of collapse, and eventually went on to complete recovery. Dr. Hunter had not expected much benefit from the tapping, since in previous cases the needle had entered the intestine and let out the gas only from a limited portion of the gut. In the present case the tube happened to penetrate only into the peritoneal cavity, from whence the gas had been unable to escape. This was the only instance he had met with in which recovery had followed puncture for tympanites. The gas which escaped was not offensive.

Dr. Nicoll said that several years ago he saw a woman, much reduced in general health, suffering from a pelvic abscess which had discharged into the vagina. She was suffering from intense dyspnœa, the result of tympanites. As a means of rendering her condition more comfortable, he punctured the abdomen in several places with a slender aspirator needle, and much fœtid gas escaped, greatly to the relief of the patient, who then went on to complete recovery. Doubtless the intestine had been punctured, as the gas was of a foul odor.

Dr. Murray referred to a case in Bellevue Hospital, in which, some years ago, the abdomen was punctured for tympanites developed after an operation. A large amount of odorless gas escaped, and the patient got well.

The Chairman thought that in a large number of cases we could not tell whether the needle entered the intestine or only the peritoneal cavity. He had heard it stated that such punctures, even if they proved of no benefit, could do no harm, for, so long as one punctured living tissue, the opening would close again immediately on withdrawing the needle, particularly if the needle was small. This, however, was a mistake, especially when we had to do with cases of peritonitis. In healthy tissue a small hole would close immediately on withdrawing the needle, because of the elasticity of the tissue; but when one had to deal with a peritonitis in which the intestine was much inflated and its wall anatomically changed by an œdematous effusion into its muscular tissue, we found tissues which were no longer normal—tissues from which, at least in some cases, the elasticity had disappeared; and in such cases he had found, at post-mortem examination, holes still remaining patent which had been made by the hypodermic needle, and through which fæces had escaped into the abdominal cavity. In such cases the intestine was in a paralytic condition, and punctures with the hypodermic needle might prove dangerous. He did not say that they were dangerous in all cases, but that they were in some he had had opportunity to verify at autopsies.

### DOUBLE OOPHORITIS A CAUSE OF ABSENCE OF MENSTRUATION.

Dr. Jacobi said he happened to be present when an autopsy was made upon two bodies in the dead-house of the Bellevue Hospital that afternoon, one of the cases being that of a girl, seventeen years of age, regarding whose sexual history nothing further was known than that she had never menstruated. He observed that both ovaries seemed to be somewhat enlarged, the tissue being unusually white, and the general appearance that of ovaries affected with chronic inflammation. He asked if this was recognized as a frequent cause of absent menstruation. The body was well-developed.

### DISTRIBUTION OF THE DIPHTHERITIC MEMBRANE.

The second case to which Dr. Jacobi referred was that of a child which had died after tracheotomy for diphtheria. It had lived two days and a half after the operation. There was slight broncho-pneumonia of both lower lobes. There was a small membrane behind the tonsils, which, probably, could not have been seen during life, the tonsils hiding the membrane entirely. The lower face of the epiglottis was completely covered with the false membrane, while the upper, as was usual in such cases, was entirely free from the exudation. The lower larynx and trachea were covered by the membrane. In the trachea was a peculiarity which

Dr. Jacobi had very rarely observed. In this locality, as a rule, we found at the post-mortem the pseudo-membrane entire, and there was so much mucus present that the membrane could easily be peeled off, and the cases were rare in which even the true diphtheritic membrane was so deeply imbedded in this situation that it could not be removed. In the present instance a part of the membrane seemed to be lying loose on the surface, and could easily be removed, while beneath was another portion firmly attached to the mucous and submucous structures, which could not be removed without taking with it the underlying tissue. The case illustrated a statement which he had made on more than one occasion, that in the same case we might be able to raise the false membrane at one point and not at all at a point a centimetre distant. The same membrane extended down, probably, into the bronchi of the third order.—*N. Y. Med. Jour.*

